# Seroprevalence of *Toxoplasma gondii* infection in Liaoning cashmere goat from northeastern China

**DOI:** 10.1051/parasite/2014023

**Published:** 2014-05-21

**Authors:** Peng Xu, Xia Li, Ling Guo, Bing Li, Jun Wang, Di Yu, Quan Zhao, Xiao-Gang Liu

**Affiliations:** 1 College of Animal Husbandry and Veterinary Medicine, Liaoning Medical University Jinzhou Liaoning Province 121001 P.R. China; 2 College of Animal Science and Technology, Jilin Agricultural University Changchun Jilin Province 130118 P.R. China; 3 Jinzhou Center for Animal Disease Control and Prevention Jinzhou Liaoning Province 121004 P.R. China

**Keywords:** *Toxoplasma gondii*, seroprevalence, Liaoning cashmere goat, indirect haemagglutination assay

## Abstract

In the present study, serum samples from 650 goats were collected from five counties between May and June 2012 and antibodies to *Toxoplasma gondii* were detected by indirect haemagglutination assay; 58 (9%) had antibodies to *T. gondii* with antibody titres of 1:64 to 1:1024. Seropositive samples were distributed in all five counties: seroprevalences in Kuandian county (15%, 21/139, 95% confidence interval [CI] 9–21%) were statistically different from the four other counties (Gaizhou, Huanren, Xiuyan and Liaoyang), and the seroprevalence difference between Xiuyan county (12%, 15/127, 95% CI 6–17%) and two other counties (Huanren, Liaoyang) was significantly different (*P* < 0.05). To our knowledge, this is the first report of the seroprevalence of *T. gondii* exposure in Liaoning cashmere goat in China. Our results indicated that Liaoning cashmere goat could be a potential reservoir for the transmission of *T. gondii* in Liaoning Province.

## Abstract (fr)

Dans cette étude, des échantillons de sérum de 650 chèvres ont été recueillis dans cinq comtés entre mai et juin 2012 et des anticorps à *T. gondii* ont été détectés par un test d’hémagglutination indirect; 58 (9 %) avaient des anticorps contre *T. gondii* avec des titres d’anticorps de 1:64 à 1:1024. Les échantillons séropositifs étaient distribués dans les cinq comtés et la séroprévalence dans le comté de Kuandian (15 %, 21/139, intervalle de confiance à 95 % [IC] 9–21 %) était statistiquement différente des quatre autres comtés (Gaizhou, Huanren, Xiuyan, Liaoyang), et la différence de séroprévalence du comté de Xiuyan (12 %, 15/127, IC à 95 % 6–17 %) et de deux autres comtés (Huanren, Liaoyang) était significativement différente. À notre connaissance, ceci est le premier rapport sur la séroprévalence d’exposition à *T. gondii* chez les chèvres cachemire du Liaoning en Chine. Nos résultats indiquent que la chèvre cachemire pourrait être un réservoir potentiel pour la transmission de *T. gondii* dans la province de Liaoning.

## Introduction

*Toxoplasma gondii* is a ubiquitous, apicomplexan parasite in warm-blooded animals and humans worldwide. Humans can be infected by ingesting raw or undercooked meat with tissue cysts, and consuming food or drink contaminated with oocysts [2]. Infection of goats with *T. gondii* may cause early embryonic death and resorption, foetal death and mummification, abortion, stillbirth and neonatal death [2], and thus can be responsible for heavy economic losses. Infection of production animals with *T. gondii* also has implications for public health since consumption of undercooked meat infected with the parasite can facilitate zoonotic transmission [2].

Liaoning cashmere goat is an excellent breed producing high-quality cashmere. To our knowledge, there is lack of information on *T. gondii* infection in Liaoning cashmere goat. The present study was conducted to investigate the seroprevalence of *T*. *gondii* infection in Liaoning cashmere goat from northeastern China and to explore the potential risks for human infection.

## Materials and methods

Liaoning Province is located in northeastern China, and borders the Yellow Sea (Korea Bay) and the Bohai Gulf in the south, North Korea in the southeast, Jilin Province to the northeast, Hebei Province to the west and Inner Mongolia to the northwest. The Yalu River marks the border between North Korea and the Chinese provinces of Jilin and Liaoning. It empties into the Korea Bay between Dandong (Liaoning) and Sinŭiju (North Korea). Liaoning Province has an area of 145,900 km^2^ and a population of approximately 44 million. The area has a temperate monsoon climate with abundant sunshine, a long winter hot summer, and a short spring and autumn. The annual average temperature is 7–11 °C, with a highest temperature of 40 °C and a lowest temperature of −30 °C.

Blood samples were collected from 650 goats via a jugular vein in Liaoning Province (40°28′–41°27′ N, 122°35′–125°35′ E), including Gaizhou, Huanren, Kuandian Xiuyan and Liaoyang in May and June 2012. Whenever possible, data regarding the geographic origin, age and gender of each animal were collected (Table 1). Blood samples were centrifuged (3000 rpm) for 5 min and stored at −20 °C until use.

**Table 1.** General characteristics of the 650 goats studied and seroprevalence of *T. gondii* infection^a^ in Liaoning Province, northeastern China.

Characteristics	No. examined	No. positive	Prevalence, % (95% CI)	*P*
Age (year)				
yr ≤ 1	34	1	2.94 (0.00–8.62)	0.11
1 < yr ≤ 2	87	5	5.75 (0.86–10.64)	
2 < yr ≤ 3	168	11	6.55 (2.81–10.29)	
3 < yr ≤ 4	239	23	9.62 (5.88–13.36)	
4 < yr ≤ 5	95	14	14.74 (7.61–21.86)	
5 < yr	27	4	14.81 (1.42–28.21)	
Gender				
Male	389	33	8.48 (5.91–11.71)	0.63
Female	261	25	9.58 (6.01–13.81)	
Location (County)				
Gaizhou	195	13	6.67 (3.17–10.17)	0.01
Huanren	86	5	5.81 (0.87–10.76)	
Xiuyan	127	15	11.81 (6.20–17.42)	
Kuandian	139	21	15.11 (9.15–21.06)	
Liaoyang	103	4	3.88 (0.15–7.61)	
Total	650	58	8.92 (6.73–11.11)	

a Difference considered significant when *P* value < 0.05.

Antibodies to *T. gondii* were determined in sera using an indirect haemagglutination antibody (IHA) test with a commercially available kit (Lanzhou Veterinary Research Institute, Chinese Academy of Agricultural Sciences, Lanzhou, Gansu Province, China) according to the manufacturer’s instructions [6]. In brief, sera were added to 96-well V-bottomed polystyrene plates, and diluted in a fourfold series from 1:4 to 1:2048. The plates were shaken for 2 min and then incubated at 37 °C for 2 h without shaking. The test was considered positive when a layer of agglutinated erythrocytes was formed in wells at dilutions of 1:64 or higher, and positive and negative controls were included in each test. The cut-off value of 1:64 was used according to the national standard (GB/T 18448.2-2008) of China for detection of *T. gondii* antibodies in humans and animals.

Differences in seroprevalence of infected goats and among associated factors were analysed using Fisher’s exact test in SAS statistical software (Version 9.3; SAS Institute Inc., Cary, NC, USA); 95% confidence intervals (CI) are given. Differences between levels within factors and interactions were considered to be statistically significant and highly significant when *P* < 0.05 and *P* < 0.01, respectively.

All animals were handled in strict accordance with good animal practice according to the Animal Ethics Procedures and Guidelines of the People’s Republic of China, and the study was approved by the Animal Ethics Committee of Liaoning Medical University.

## Results and discussion

Antibodies to *T. gondii* were found in 58 (8.9%) of 650 goats in titres of 36 sera with a titre of 64, 11 of 128, 6 of 256, 3 of 512 and 2 of 1024.

The results of the univariate analysis are shown in Table 1. 8.92% of the 650 tested Liaoning cashmere goats were seropositive for *T. gondii* by IHA, which is lower than the percentage reported in Shaanxi (IHA, 14.1%) [6], Guangxi (IHA, 41.2%) [4] and Qinghai (IHA, 17.29%) [3], but higher than that reported in Shanghai (IHA, 2.39%) [1], Heilongjiang province (IHA, 3.8%) [5] and Tibet (IHA, 5.7%). This phenomenon was possibly due to the special geographic environment as the origin of three rivers (Fuer, Xiongyue and Yalu rivers) and a large number of wild animals as the source for *T. gondii* infection. The reasons may be that wild animals and Liaoning cashmere goats live together in the same pasture, and free-grazing Liaoning cashmere goats have more chances to ingest *T. gondii* oocysts than captive goats in other regions.

Different *T. gondii* seroprevalences in Liaoning cashmere goat in different countries and regions may be due to the different serological tests used and different sources of Liaoning cashmere goat. *T. gondii* infection is probably more prevalent in warm and humid areas than in cold and dry regions. This is probably related to conditions relating to the survival of oocysts in the environment.

The logistic regression showed that all the factors (sex, age and location) reported in the present study affected the prevalence of infection (Table 1). The seroprevalence in female goats (9.58%) was higher than that in males (8.48%), and the difference was not statistically significant (*P* > 0.05). Seroprevalence in goats increased progressively with age, ranging from 2.94% to 14.81%, with the highest of 14.81% in Liaoning cashmere goats which were >5 year old, but the seroprevalences were not statistically significantly different among the different age groups (*P* > 0.05). The varied seroprevalence in different age groups suggests the possibility of horizontal transmission in the investigated herds. Though the sampling size was too small to make a conclusive statement, the results of the present study and other surveys indicate that a significant population of goats is exposed to *T*. *gondii* infection, and infected goats could be a major infection source for humans in China.

## Conclusions

The present study showed that *T. gondii* infection is prevalent in Liaoning cashmere goats of all age ranges in northeastern China, which may represent a potential source of human infection with *T. gondii*. Therefore, it is necessary to implement integrated strategies, including efficient management measures to prevent and control *T. gondii* infection in cashmere goat in the study region.
